# Differential Impact of Allergen Sensitization on Asthma Development in High-Risk Japanese Infants with Atopic Dermatitis: The Possible Role of Peanut Sensitization

**DOI:** 10.31662/jmaj.2025-0301

**Published:** 2025-12-05

**Authors:** Sayaka Hamaguchi, Kotaro Umezawa, Kenji Toyokuni, Yasuhito Yamamoto, Tatsuki Fukuie, Yukihiro Ohya, Kiwako Yamamoto-Hanada

**Affiliations:** 1 Allergy Center, National Center for Child Health and Development, Tokyo, Japan; 2 Department of Pediatrics, Tokyo Metropolitan Hiroo Hospital, Tokyo, Japan; 3 Department of Occupational and Environmental Health, Graduate School of Medical Sciences, Nagoya City University, Nagoya, Japan; 4 Division of General Allergy, Bantane Hospital, Fujita Health University, Aichi, Japan

**Keywords:** asthma, atopic dermatitis, egg white, House dust mite, IgE sensitization, peanut

## Abstract

**Introduction:**

The atopic march describes the progression from early eczema to food allergies and asthma. In Japan, early-onset atopic dermatitis (AD) is linked to allergic diseases, but the role of peanut sensitization―a known asthma risk factor in Western populations―remains uncertain because of its lower prevalence. This study aimed to evaluate whether peanut sensitization predicts asthma development in Japanese children with AD and to compare its predictive value with other common allergens.

**Methods:**

We conducted a retrospective cohort study of 203 children under age two with physician-diagnosed AD who underwent simultaneous measurement of specific immunoglobulin E to egg white, peanut, and house dust mite at a tertiary Allergy Center in Tokyo. Participants were followed until age six. Sensitization was categorized as none, mono (one allergen), or oligo (≥2 allergens). Associations with asthma development were evaluated using multivariate logistic regression and decision tree analysis.

**Results:**

Asthma developed in 32.0% of participants. Peanut sensitization was significantly more common in the asthma group (53.8% vs. 29.7%, p = 0.001), as were oligo-sensitization and allergic rhinitis. Decision tree analysis identified peanut sensitization as the most influential predictor, with an 83% asthma incidence among children with both peanut sensitization and rhinitis. Logistic regression confirmed peanut sensitization as an independent risk factor (adjusted odds ratio: 2.74, 95% confidence interval: 1.44-5.24).

**Conclusions:**

Peanut sensitization in infancy strongly predicts asthma in Japanese children with AD. Early allergen-specific sensitization profiling may help identify high-risk children and support targeted asthma prevention strategies.

## Introduction

The atopic march typically starts with atopic dermatitis (AD) (eczema) in infancy, followed by food allergies, and later, respiratory allergies such as allergic rhinitis and asthma. This sequence reflects a pattern in which early skin barrier dysfunction and immune sensitization to allergens can predispose individuals to subsequent allergic diseases ^(1), (2), (3)^.

In Japan, studies have shown that children with early eczema are at a higher risk of developing food allergies and asthma. For instance, a study involving Japanese children found that early sensitization to inhalant allergens such as house dust mites (HDMs) and Japanese cedar pollen increased the risk of developing asthma and food allergies ^(4)^. Polysensitization, particularly to inhalant allergens, has been well established as a risk factor for the development of asthma in numerous studies ^(5), (6)^. Moreover, early-life sensitization to food allergens during infancy has also been associated with an increased risk of developing asthma ^(7)^.

We recently demonstrated that in infants with AD, the onset and peak of allergen sensitization vary depending on the type of allergen. Sensitization to egg typically peaks around the time of weaning, whereas sensitization to peanuts, tree nuts, and HDM peaks approximately one year later ^(8)^.

In young patients with asthma, sensitization to peanut has been reported to be associated with elevated levels of inflammatory markers such as fractional exhaled nitric oxide, a key indicator of airway inflammation ^(9)^. A longitudinal study demonstrated that peanut sensitization was associated with markers of both airway and systemic inflammation, particularly among younger individuals. These findings suggest that peanut sensitization may serve as a risk factor for the development of airway inflammation ^(10)^. Peanut allergy prevalence is high in the West, and its association with asthma has been reported. However, peanut sensitization is less common in Japanese individuals because of racial differences. In this study, we aimed to clarify whether peanut sensitization predicts the development of asthma in a high-risk Japanese population with AD, which represents a key group in the allergic march, similar to findings reported in Western populations.

The aim of this study was to determine whether peanut sensitization is a risk factor for asthma in high-risk Japanese populations, and whether its association with asthma is stronger compared to other allergens in children with AD.

## Materials and Methods

### Study design and setting

This retrospective cohort study utilized electronic medical record data from patients who were hospitalized or received outpatient treatment at the National Center for Child Health and Development (NCCHD) in Tokyo, a World Allergy Organization Center of Excellence, between June 2013 and June 2023. The study was approved by the Ethics Committee of the NCCHD (approval number: 2023-083). Informed consent was obtained via an opt-out method on the institution’s website.

### Participants

Eligible participants were children under two years of age with AD who visited the Allergy Center. The diagnosis of AD was defined as physician-diagnosed AD recorded in the electronic medical records.

### Variables

The variables evaluated included patient demographics (age, sex), comorbidities (e.g., allergic rhinitis), and laboratory data (specific immunoglobulin E [IgE] levels for egg white, peanut, and HDM).

Two separate models were constructed to assess the associations with asthma development.

Model 1: included sex, sensitization to specific IgE (egg white, peanut, and HDM), and the presence of rhinitis by age six years.

Model 2: included sex, oligo-sensitization (defined as sensitization to two or more allergens), and rhinitis by age six years.

Other common allergens, such as tree nuts, soy, and pet dander, were not included because specific IgE to these allergens was not consistently measured in infancy at our center. To ensure comparability, we restricted the analysis to allergens for which specific IgE was tested simultaneously in all participants (egg white, peanut, and HDM).

### Database and evaluation methods

Data were extracted from the electronic medical records of the NCCHD. Patients treated at the Allergy Center between June 2013 and June 2023 were screened by the Medical Record Information Management Department using the following criteria:

1. Diagnosis code for AD

2. History of steroid prescription

3. History of visits to an allergist

4. Age under two years at the time of allergy testing

From the eligible records, the following data were extracted: sex, age, comorbid allergic diseases (e.g., rhinitis up to age six), specific IgE levels (egg white, HDM, peanut), and asthma onset up to age six. Specific IgE levels were measured using the ImmunoCAP system (Thermo Fisher Scientific, Inc., Waltham, MA, USA). A value >0.35 IU/mL was considered indicative of sensitization.

Asthma and allergic rhinitis were diagnosed by pediatricians or allergy specialists. Asthma was defined as a diagnosis made after the date of specific IgE testing in children under six years of age.

Sensitization status was categorized as follows:

・None: No sensitization to any of the three allergens

・Mono: Sensitization to only one allergen

・Oligo: Sensitization to two or more allergens (egg white, peanut, or HDM)

### Statistical methods

Patient characteristics were compared according to the presence or absence of asthma using the χ^2^ test, performed with GraphPad Prism version 9.0 (GraphPad Software, La Jolla, CA, USA). To investigate the relationship between specific IgE sensitization and the development of asthma, both decision tree analysis and multivariate logistic regression were employed.

Due to multicollinearity, specific IgE values and the total number of sensitizations (defined as the count of positive sensitizations to peanut, egg white, and HDM) were analyzed in separate models. Decision tree analysis was conducted using the Classification and Regression Trees algorithm implemented in Python. This method hierarchically identifies the most influential predictors of asthma onset and presents them in an interpretable, tree-like structure of decision rules. This approach is particularly valuable in clinical research for uncovering complex interactions among variables and for generating hypotheses regarding key risk factors in high-risk pediatric populations ^(11)^. Decision tree analysis was conducted using two models. The first model included sex, sensitization to specific IgE (egg white, peanut, and HDM), and the presence of rhinitis by age six years. The second model included sex, oligo-sensitization (sensitization to two or more allergens), and rhinitis by age six years.

Multivariate logistic regression was also performed using GraphPad Prism to quantify the independent associations between specific IgE sensitizations and asthma development, adjusting for potential confounders. Logistic regression analyses were conducted using two separate models. The first model included sex, sensitization to specific IgE (egg white, peanut, and HDM), and the presence of rhinitis by age six years. The second model included sex, oligo-sensitization, and rhinitis by age six years. All variables within each model were mutually adjusted.

Specific IgE values below 0.1 IU/mL were treated as 0.1, and values above 100 IU/mL were capped at 100 IU/mL. A p < 0.05 was considered statistically significant. There were no missing data in the dataset.

This dual-approach―combining interpretable machine learning (decision tree) with traditional statistical modeling (logistic regression)―enhances the robustness of the findings and provides both exploratory and confirmatory insights into the role of allergen sensitization in asthma development.

## Results

### Participant/descriptive data: characteristics of participants

**Figure 1.**
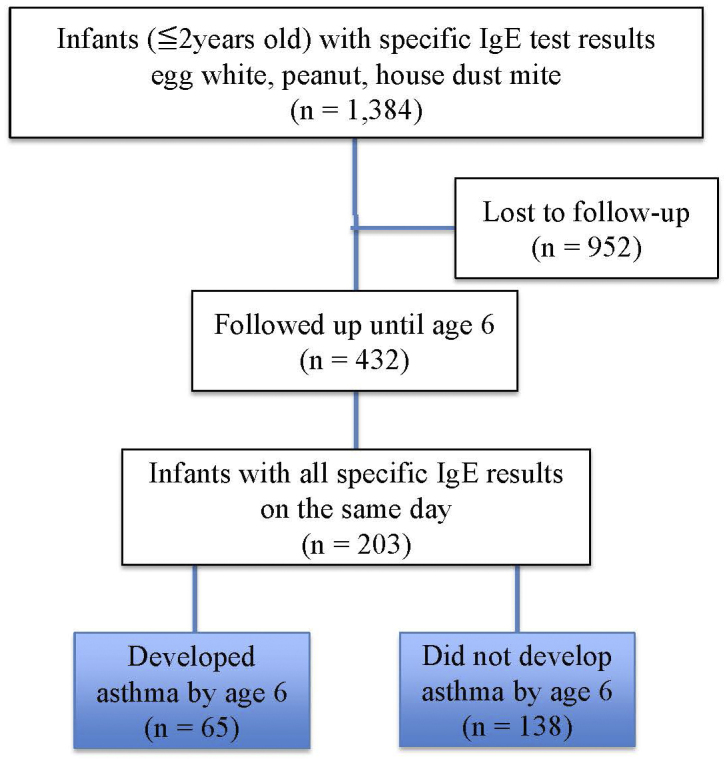
Study design. The number of children with atopic dermatitis who have specific IgE (HDM, egg white, peanut) values under two years of age was 1,384. Of these, 203 were followed up to age six years, and all specific IgE: egg white, peanut, and HDM were measured on the same day. 65 (32.0%) had asthma onset by age six years. HDM: house dust mite, IgE: immunoglobulin E.

The number of children with AD who have specific IgE (HDM, egg white, peanut) values measured under two years of age was 1,384. Of these, 203 were followed up to age six years, and all specific IgE values (egg white, peanut, and HDM) were measured on the same day. Sixty-five (32.0%) had asthma onset by age six years. (Figure 1) Compared to the group without asthma, the group with asthma onset had higher rates of oligo-sensitization (67.7% vs. 38.4%, p < 0.001), peanut sensitization (53.8% vs. 29.7%, p = 0.001), HDM sensitization (40.0% vs. 23.2%, p = 0.013), and rhinitis by age 6 (16.9% vs. 6.9%, p = 0.02). The median age of asthma onset was three years (Table 1).

**Table 1.** Patient Characteristics.

Characteristics	No asthma	Asthma	p Value	
Patient, n	138	65		
Male, n (%)	96 (69.6%)	46 (70.8%)	0.861	ns
Age of asthma onset, years, median (IQR)		3 (2-4)		
				
Age at time of blood test, months, median (IQR)	12 (9–18)	18 (12–20.3)		
Specific IgE sensitization (%)^†^				
Egg white	114 (83.6%)	59 (90.8%)	0.126	ns
Peanut	41 (29.7%)	35 (53.8%)	0.001	**
HDM	32 (23.2%)	26 (40.0%)	0.013	*
Number of specific IgE sensitizations, n (%)				
Oligo	53 (38.4%)	44 (67.7%)	<0.0001	****
Mono	65 (47.1%)	17 (26.1%)	0.004	**
None	21 (15.2%)	4 (6.2%)	0.038	*
				
Allergic rhinitis (%)	9 (6.5%)	11 (16.9%)	0.02	*

χ^2^ test; Statistical analysis was performed with GraphPad Prism 9.0 (La Jolla, CA, USA). *p < 0.05. **p < 0.01. ****p < 0.0001. ^†^Serum IgE was measured by ImmunoCAP: Sensitization >0.35 IU/mL. HDM: house dust mite; IgE, Immunoglobulin E; IQR: interquartile range; ns: not significant.

### Decision tree analysis

**Figure 2.**
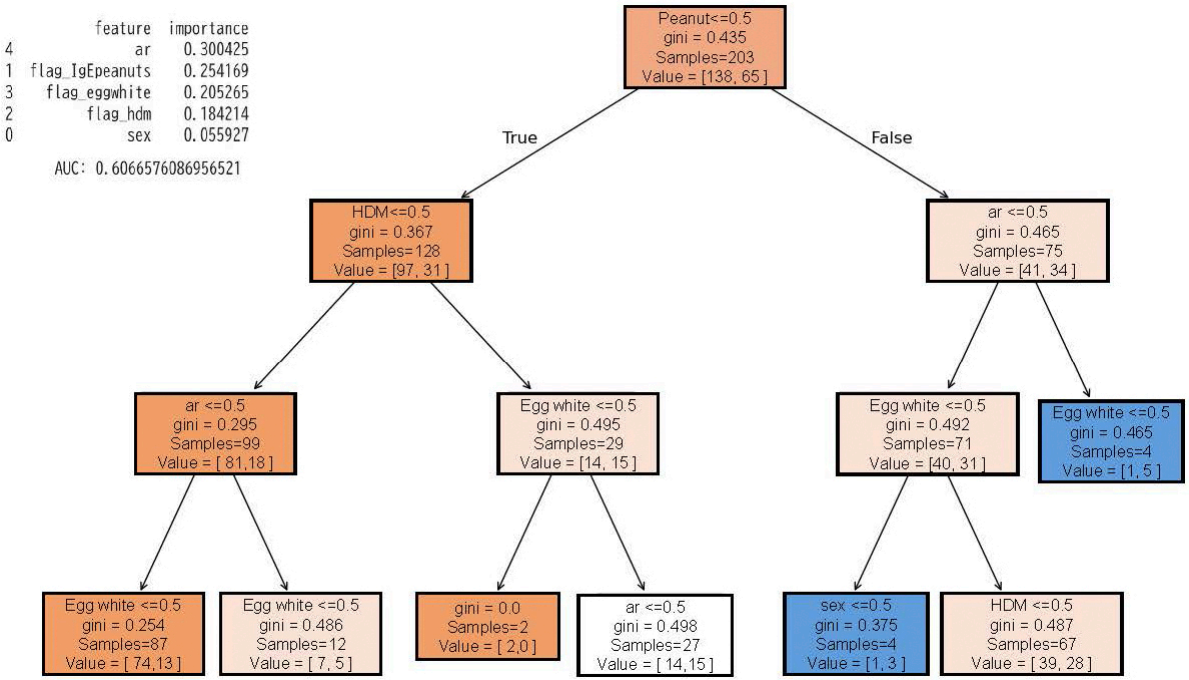
Decision tree analysis: specific IgE. Decision tree analysis for sex, sensitization to specific IgE (egg white, peanut, HDM), and rhinitis up to age six years. HDM: house dust mite, IgE: immunoglobulin E.

**Figure 3.**
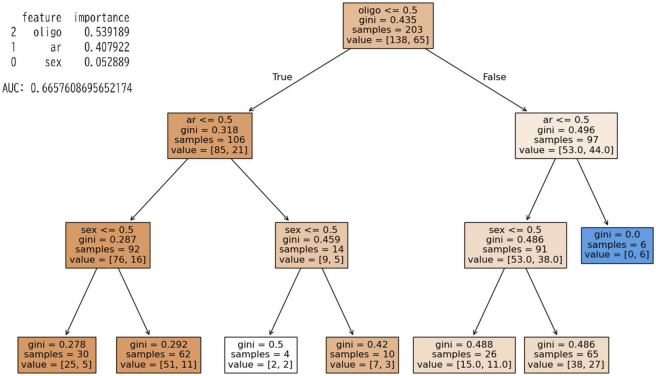
Decision tree analysis: oligo-sensitization. Decision tree analysis for sex, oligo-sensitization to specific IgE, and rhinitis up to age six years. HDM: house dust mite, IgE: immunoglobulin E.

Decision tree analysis for sex, sensitization to specific IgE (egg white, peanut, HDM), and rhinitis up to age six years showed that the highest-ranking factor was peanut sensitization (Figure 2). This population had a 32% incidence (65 asthma / 203 overall), and when divided by the presence of sensitization to peanut-specific IgE, the incidence of asthma in the peanut-sensitized group was 45%. In the case of peanut sensitization and rhinitis, the population had an 83% incidence of asthma. The importance values were as follows: Allergic rhinitis(AR): 0.30, peanuts sensitization: 0.25, egg sensitization: 0.21, and HDM: 0.18. The accuracy of this model was an area under the curve (AUC) of 0.61. (Figure 2) Similarly, when analyzing sex, oligo-antigen sensitization to specific IgE, and rhinitis up to the age of six years, oligo-sensitization was the highest predictor. The importance values were as follows: oligo-sensitization: 0.54 and AR: 0.4. The accuracy of this model was an AUC of 0.67. (Figure 3)

### Multiple logistic regression analysis

**Figure 4.**
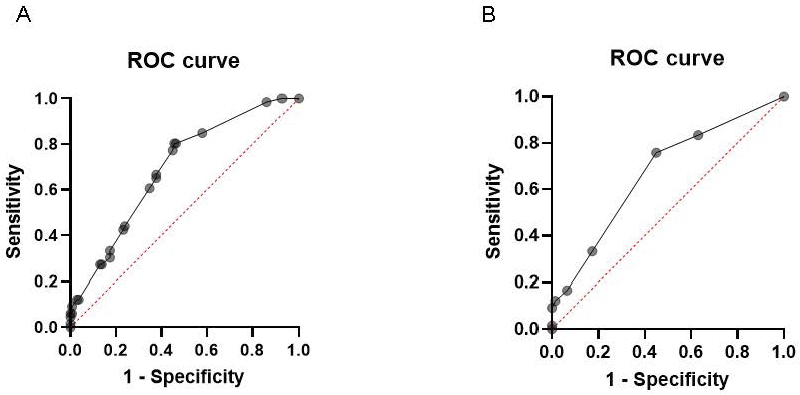
The accuracy of these models. A. AUC for sex, sensitization to specific IgE (egg white, peanut, HDM), and rhinitis by age 6 years. B. AUC for sex, oligo-sensitization to specific IgE, and rhinitis by age six years. AUC: area under the curve, HDM: house dust mite, IgE: immunoglobulin E.

In the first model, multiple logistic regression analysis was performed to evaluate the associations of sex, sensitization to specific IgE (egg white, peanut, and HDM), and rhinitis by age six years with asthma. The adjusted odds ratios (aORs) and 95% confidence intervals (CIs) were as follows: peanut sensitization (aOR, 2.75; 95% CI: 1.44-5.24), egg white sensitization (aOR, 1.49; 95% CI: 0.55-4.07), HDM sensitization (aOR, 1.87; 95% CI: 0.95-3.67), and rhinitis (aOR, 4.24; 95% CI: 1.55-11.6). Among these, peanut sensitization and rhinitis by age six years were significantly associated with an increased risk of asthma, whereas sensitization to egg white and HDM showed no statistically significant associations (Table 2). The accuracy of this model was an AUC of 0.6904 (p < 0.0001) (Figure 4A).

**Table 2.** Multiple Logistic Regressions.

Variables	aORs	95% Cl
The association of specific IgE with asthma		
Sex	0.90	0.45-1.80
Specific IgE sensitization^†^		
Egg white	1.49	0.55-4.07
Peanut	2.75	1.44-5.24
HDM	1.87	0.95-3.67
Allergic rhinitis	4.24	1.55-11.62
The association of oligo-sensitization with asthma		
Sex	0.97	0.49-2.00
Oligo sensitization	4.13	2.17-8.21
Allergic rhinitis	4.40	1.60-12.55

Multiple logistic regression analyses were conducted using two separate models. The first model included sex, sensitization to specific IgE (egg white, peanut, and HDM), and the presence of rhinitis by age six years. The second model included sex, oligo-sensitization, and rhinitis by age six years. All variables within each model were mutually adjusted. Multiple logistic regressions were performed with GraphPad Prism 9.0 (La Jolla, CA, USA). ^†^Serum IgE was measured by ImmunoCAP: Sensitization >0.35 IU/mL. aOR: adjusted odds ratio; CI: confidence interval; HDM: house dust mite; IgE: immunoglobulin E.

In the second model, multiple logistic regression analysis was conducted to assess the associations of sex, oligo-sensitization, and rhinitis by age six years with the risk of developing asthma. The aORs and 95% CI were as follows: oligo-sensitization (aOR, 4.13; 95% CI: 2.17-8.21) and rhinitis by age six years (aOR, 4.40; 95% CI: 1.60-12.6) (Table 2). The accuracy of this model was AUC 0.6691 (p < 0.0001) (Figure 4B)

## Discussion

This study offers novel and clinically relevant insights into how different allergen sensitizations influence asthma development in a high-risk pediatric population with AD in Japan. Notably, peanut sensitization emerged as a particularly strong and independent predictor of asthma onset by age six, surpassing the predictive strength of more commonly sensitized allergens such as egg white and HDM.

While egg white sensitization was more prevalent in this cohort, peanut sensitization was more strongly associated with asthma, as demonstrated by both decision tree analysis and multivariate logistic regression. This finding is particularly striking given that peanut is a relatively less common allergen in Japan―ranked fifth among pediatric food allergies―compared to Western countries, where it is a leading cause of food allergy ^(12), (13)^. The strength of this association suggests that peanut sensitization may represent a distinct immunological pathway with a higher risk for respiratory outcomes.

The use of decision tree analysis, a machine learning method that identifies hierarchical relationships among variables, adds methodological novelty and interpretability to this study ^(11)^. It revealed that children with both peanut sensitization and allergic rhinitis had an asthma incidence as high as 83%, highlighting a synergistic risk. In contrast, egg white and HDM sensitizations showed weaker associations, suggesting that not all allergens confer equal risk for asthma, and that the type of allergen matters.

These findings are consistent with recent international cohort studies that have shown varying asthma risks depending on the type and timing of sensitization. For example, the Canadian Healthy Infant Longitudinal Development study found that early food allergen sensitization was more predictive of asthma than inhalant sensitization ^(7)^. Similarly, studies from Germany and the U.S. (e.g., Childhood Origins of Asthma study) have identified specific inhalant allergens such as Der p 1, Der p 23, and dog dander as high-risk sensitizers ^(6), (14)^. However, few studies have directly compared food allergens such as peanut and egg white in relation to asthma risk ^(15)^, making our findings particularly valuable.

The biological mechanisms underlying these differences may involve distinct sensitization routes. Peanut proteins such as Ara h 1 and Ara h 2 have been shown to disrupt bronchial epithelial barriers and may facilitate trans-airway sensitization, potentially explaining the stronger link to asthma ^(16), (17), (18)^. In contrast, egg white sensitization may be more closely associated with percutaneous sensitization, particularly in the context of skin barrier dysfunction and co-sensitization to skin fungi like Malassezia ^(8), (19)^.

Regarding this difference in the influence of different allergens, egg white had a higher rate of sensitization than peanut in this study, and it is possible that peanut sensitization is more strongly associated with oligo-sensitization. Nevertheless, in our previous research on children with AD under two years of age, we observed a later onset and lower overall sensitization rates for peanut and HDM compared to egg white. Notably, the sensitization rates for peanut and HDM were nearly identical ^(8)^. These findings suggest that the association between peanut sensitization and asthma is not merely a consequence of oligo-sensitization, but may instead reflect an independent effect of peanut sensitization itself.

### Strengths and limitations

This study has several methodological strengths that enhance the validity and robustness of its findings. First, we employed a dual-analytic approach combining traditional multivariate logistic regression with decision tree analysis, a machine learning technique. This allowed us to both quantify independent associations and explore complex interactions among variables in an interpretable, hierarchical structure. The consistency of findings across these two complementary methods strengthens the credibility of our conclusions.

Second, all specific IgE measurements were conducted on the same day using a standardized and validated assay (ImmunoCAP), ensuring comparability across participants. The study population was rigorously defined using electronic medical records, and there were no missing data, eliminating the need for imputation and reducing potential bias. Furthermore, multicollinearity was addressed by analyzing individual allergen sensitizations and the number of sensitizations in separate models.

Third, the use of decision tree analysis adds methodological novelty, particularly in the context of pediatric allergy research in Japan, where such approaches remain underutilized. This technique provided clinically intuitive insights into the relative importance of different allergens, highlighting peanut sensitization as a key predictor of asthma development.

The relatively modest AUC values (0.61-0.69) indicate that, although peanut sensitization and rhinitis are significant predictors, the models are not sufficient for stand-alone clinical prediction. Rather, these findings highlight the contribution of early sensitization patterns to asthma risk and should be interpreted as supportive markers within a multifactorial clinical context.

However, several limitations should be acknowledged. As a retrospective cohort study, the analysis was limited to children who remained in follow-up until age six, which may introduce selection bias. Additionally, to ensure consistency in IgE measurement timing, we restricted the analysis to children with all three allergen-specific IgEs measured on the same day, which limited the inclusion of other potentially relevant allergens such as tree nuts or soy. Furthermore, sensitization to Japanese cedar pollen (Cryptomeria japonica), one of the most prevalent aeroallergens in Japan and a major contributor to allergic rhinitis and asthma in later childhood ^(4)^, was not assessed. It could not be included in the present analysis because sensitization rarely occurs in infancy, and specific IgE to cedar pollen is not routinely tested at this age. Nevertheless, cedar pollen sensitization represents an important factor in the atopic march, and its inclusion in future prospective studies with longer follow-up periods and broader allergen panels will be essential to fully elucidate its impact on longitudinal allergic outcomes. Finally, environmental factors such as household peanut exposure or regional allergen levels were not assessed, which may influence sensitization patterns and asthma risk. Future prospective studies incorporating environmental exposure data and a broader range of allergens are warranted to validate and expand upon these findings.

### Conclusions

Our findings highlight a distinct and clinically significant link between early peanut sensitization and asthma development in Japanese children with AD. The results emphasize that the type of allergen sensitized―not just the presence of sensitization―plays a pivotal role in asthma risk. These insights support the need for tailored early screening and intervention strategies, particularly targeting peanut sensitization, to mitigate asthma onset in this vulnerable population.

## Article Information

### Acknowledgments

Thank you to all physicians who collected blood samples to measure IgE titers. We gratefully acknowledge Hirotaka Chiba of the Department of Information Management, National Center for Child Health and Development, for his assistance with data extraction.

### Author Contributions

Conceptualized the study and contributed to the study design: Sayaka Hamaguchi. Performed data extraction and analysis and drafted the initial manuscript: Sayaka Hamaguchi. Contributed to data curation and statistical validation: Kotaro Umezawa and Tatsuki Fukuie. Provided technical and methodological support: Kenji Toyokuni and Yasuhito Yamamoto. Supervised the overall project: Yukihiro Ohya. Critically revised the manuscript for important intellectual content: Kiwako Yamamoto-Hanada. All authors reviewed and approved the final version of the manuscript.

### Conflicts of Interest

None

### IRB Approval Code and Name of the Institution

The study was approved by the Ethics Committee of the National Center for Child Health and Development (approval number: 2023-083).
